# Ionospheric and Neutrosphere Impacts on Multi-GNSS Kinematic PPP During Geomagnetic Storms: A Global Study

**DOI:** 10.3390/s26134037

**Published:** 2026-06-25

**Authors:** João P. V. Zaupa, Felipe T. L. De Souza, Lucas G. Ferreira, Henrique Y. Yamashiro, Tayná A. F. Gouveia, Daniele B. M. Alves, João F. G. Monico, Vinicius A. S. Pereira, Paulo T. Setti

**Affiliations:** 1 Department of Cartography, São Paulo State University (UNESP), Presidente Prudente 19060-080, São Paulo, Brazil; felipe.tintino@unesp.br (F.T.L.D.S.); lucas.g.ferreira@unesp.br (L.G.F.); h.yamashiro@unesp.br (H.Y.Y.); tayna.gouveia@unesp.br (T.A.F.G.); daniele.barroca@unesp.br (D.B.M.A.); galera.monico@unesp.br (J.F.G.M.); p.setti@unesp.br (P.T.S.J.); 2 Course of Agronomy, Federal University of Technology—Paraná (UTFPR), Santa Helena Campus, Santa Helena 85892-000, Paraná, Brazil; vpereira@utfpr.edu.br

**Keywords:** neutrosphere effects, PPP, ROTI, space weather

## Abstract

This work proposes a multiscale spatial and temporal approach to assess the impacts of the ionosphere and neutrosphere (neutral atmosphere including both tropospheric and stratospheric) through an independent analysis of each component on Precise Point Positioning (PPP) accuracy and stability during selected representative geomagnetic events of Solar Cycle 25. Geomagnetically quiet and disturbed days were selected using the Kp index, with 21 multi-GNSS stations distributed across latitude bands. Kinematic PPP processing was performed using APPPOLO software (v1.0) with ionosphere-free dual-frequency combinations, precise products, and robust filtering, totaling 924 solutions. Results show improvements in geometry and satellite availability with multi-GNSS, achieving discrepancies within 0–10 cm in more than 89% of the solutions. The VMF3 model confirmed the deterministic behavior of ZHD and the latitudinal variability of ZWD, with increased stability in multi-GNSS solutions. Greater degradation was observed at high latitudes under disturbed geomagnetic conditions, particularly for GPS-only processing. Residual analysis indicated elevation-dependent effects and constellation-related differences. The analysis of ionospheric irregularities using ROTI revealed that PPP degradation is strongly associated with spatial distribution and satellite geometry, with enhanced effects at high latitudes and low elevation angles.

## 1. Introduction

Absolute geodetic positioning has played a key role in scientific and technological applications that require high precision, with the Precise Point Positioning (PPP) method being widely used. This method allows centimeter-level solutions to be obtained from undifferentiated Global Navigation Satellite Systems (GNSS) observations, using accurate orbit and clock products, without the need for local reference stations [1]. However, the reliability of PPP solutions depends heavily on the proper modeling of the effects that affect the propagation of GNSS signals, especially those associated with the ionosphere and neutrosphere. In addition, solar phenomena, such as solar and geomagnetic storms, introduce significant disturbances in these media, directly impacting the accuracy and stability of position estimates [2,3].

The neutrosphere is a layer of the atmosphere that extends from the surface to about 50 km altitude (in the GNSS/PPP literature, the terms neutral atmosphere or troposphere are commonly used, but this terminology is physically incomplete) [4]. This layer influences the propagation of GNSS signals by introducing a tropospheric propagation delay. This delay is commonly represented by the zenith tropospheric delay (ZTD), whose hydrostatic component is dominant, relatively constant, and easily modeled, while the wet component exhibits high spatiotemporal variability, constituting the primary source of uncertainty [4]. This variability is associated with the heterogeneous distribution of water vapor, dynamic processes in the boundary layer, and atmospheric turbulence, resulting in fluctuations on multiple scales, ranging from seasonal patterns to high-frequency variations. In addition, horizontal anisotropies represented by tropospheric gradients (typically ~0.7–1.0 mm, reaching up to 4–6 mm in severe events) introduce directional errors in GNSS observations, especially at low elevations. The accuracy of modeling these effects depends heavily on the resolution of atmospheric models and the ability to adequately represent this variability, with the wet component being the main limiting factor in high-precision GNSS applications [5,6,7].

**Figure 1:**
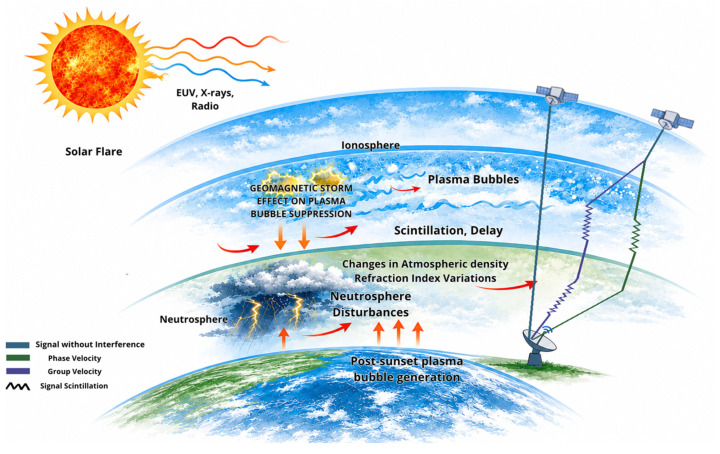
Demonstration of the effect of geomagnetic storms on GNSS signals and their propagation media. The illustrative background was generated with the assistance of ChatGPT Plus, GPT 5.1 model (available on 29 December 2025), and subsequently edited by the authors; all scientific annotations and conceptual elements were prepared and verified by the authors.

On a global scale, the propagation of GNSS signals is influenced both by phenomena related to the environment and by effects associated with the signal source. Ionospheric variability is strongly dependent on geomagnetic latitude, seasonality, and solar activity levels, and is particularly intense in equatorial regions, where the Equatorial Ionization Anomaly (EIA) is observed [8]. Additionally, the South American Magnetic Anomaly (SAMA) contributes to increased exposure to energetic radiation, enhancing ionospheric disturbances [9]. These processes are modulated by the approximately 11-year solar cycle, with the current Solar Cycle 25 characterized by an increase in the frequency of extreme solar events [8]. Solar phenomena, particularly solar flares and geomagnetic storms, affect the ionosphere through a sudden increase in electromagnetic radiation in the X-ray and extreme ultraviolet bands, resulting in rapid variations in electron density [10,11]. These effects manifest themselves through changes in Total Electron Content (TEC) and the intensification of ionospheric irregularities that cause scintillations, which can degrade GNSS signals and compromise the quality of the PPP solution (Figure 1) [10,11].

Figure 1 schematically shows the effects of solar activity and atmospheric layers on the propagation of GNSS signals, highlighting the ionosphere and neutrosphere. Solar radiation intensifies variations in electron density, favoring the formation of plasma bubbles and causing phenomena such as signal delay, dispersion, and scintillation, affecting phase and group velocity differently (same intensity, but opposite signs). Additionally, the neutrosphere contributes to non-dispersive delays associated with variations in the refractive index, highlighting the importance of correct atmospheric modeling for high-precision GNSS applications.

The impacts of atmospheric and solar effects on the performance of GNSSs have been extensively investigated in the literature. Recent studies analyze PPP degradation under disturbed ionospheric conditions [12], the direct effects of solar flares on GNSS signals [11], as well as the need for multiscale approaches to characterize these impacts [13]. In addition, extreme Space Weather events have demonstrated the potential to compromise critical GNSS time-based applications [14].

Given this context, this study aims to develop a multi-scale spatial and temporal approach to characterize the combined impacts of the ionosphere and neutrosphere on the accuracy and stability of multi-GNSS PPP during selected representative geomagnetic events of Solar Cycle 25.

## 2. Methodology

This section presents the methodological procedures adopted in this study. First, it describes the criteria used to select the days of interest, distinguishing between geomagnetically disturbed and calm periods based on the Kp index. Next, it details the process of choosing stations, considering data availability, multi-GNSS compatibility, and global distribution by geomagnetic latitudes. Finally, the PPP processing configurations and the strategies employed for the adjustment, smoothing, and quality control of the observations are presented.

### 2.1. Date Selection

**Figure 2:**
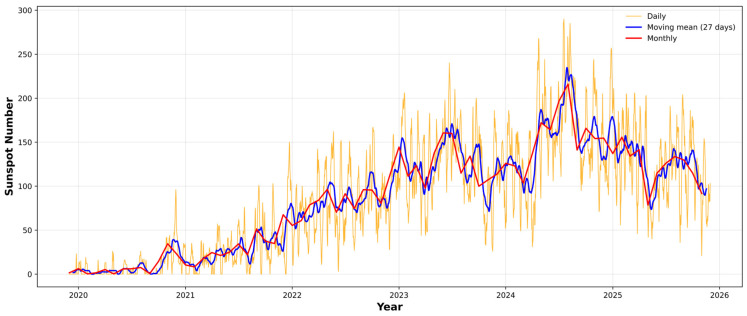
Daily and monthly Sunspot Number from December 2019 to November 2025 [15].

The days analyzed were selected from the Solar Cycle 25 period, spanning from its onset in December 2019, characterized by low levels of solar activity, to late 2025, after the period of increased solar activity associated with the cycle’s maximum. Figure 2 illustrates the temporal evolution of the Sunspot Number throughout Solar Cycle 25, demonstrating the variability of solar activity over the study period.

As a basis for the study, the Kp index (Planetary K-index) was used as a metric to define two distinct sets of interest: the set of geomagnetically disturbed days (HIGH) and the set of geomagnetically calm days (LOW), to enable a more detailed comparison between these two sets in the context of PPP.

**Figure 3:**
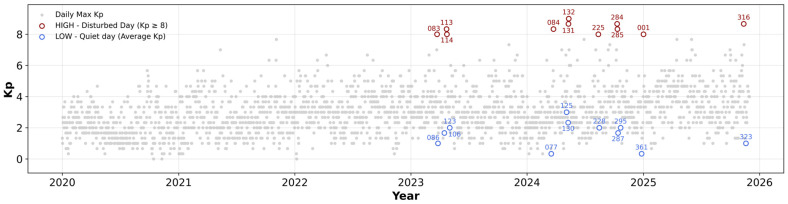
Selected geomagnetically calm and disturbed days [16,17]. Marker labels indicate day of year (DOY).

For the selection of geomagnetically disturbed days, a threshold value of Kp ≥ 8 was adopted, which includes severe (G4) and extreme (G5) geomagnetic storms, according to the classification used by the National Oceanic and Atmospheric Administration (NOAA) [16,17]. These high Kp values are associated with significant disturbances in the Earth’s geomagnetic field and highly dynamic ionospheric conditions, potentially capable of impacting the performance of satellite navigation systems. For the selection of geomagnetically quiet days, three complementary criteria were adopted. The selected day had to fall within a time window of up to 10 days before or 10 days after the respective geomagnetically disturbed day. In addition, none of the eight daily observations of the Kp index should exceed Kp = 4, ensuring the absence of geomagnetic disturbances that could interfere with the results. Finally, among the candidate days that met the above criteria, the one with the lowest daily average Kp index value was selected. Figure 3 shows the geomagnetically disturbed and calm days selected after applying the criteria described above.

For each disturbed day a corresponding quiet day was selected for comparison purposes. In total, 22 representative days (11 geomagnetically disturbed and 11 geomagnetically calm days) were defined for the study based on the proposed selection criteria. The complete list of selected dates is presented in Table 1.

To validate the selection of disturbed days, the Disturbance Storm Time (Dst) index was also evaluated using data provided by the World Data Center for Geomagnetism, Kyoto [18]. The selected disturbed periods (Kp ≥ 8) coincide with significant negative values of the Dst index, as all of them are below −100 nT, which characterize strong geomagnetic storms according to [19]. For some periods, the Dst index reached values below −200 nT and even −350 nT, corresponding to severe and great storms, respectively.

**Figure 4:**
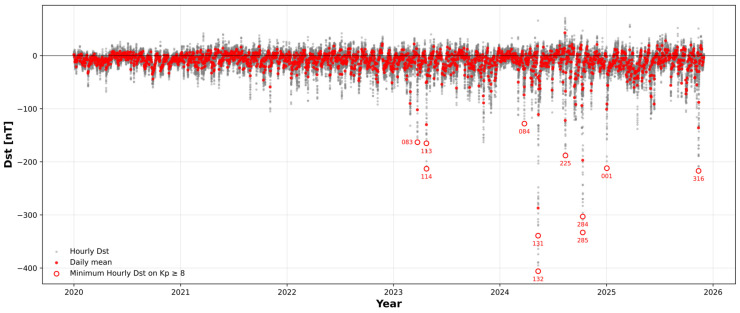
Daily and monthly variations of the Dst index during Solar Cycle 25. The highlighted dates indicate the geomagnetically disturbed days selected according to the criterion Kp ≥ 8. Marker labels indicate day of year (DOY).

The data show agreement between the Kp and Dst indexes, confirming that the selected dates correspond to periods of strong geomagnetic disturbance. Figure 4 shows the temporal behavior of the Dst index during Solar Cycle 25, highlighting the correspondence between the disturbed days selected based on the Kp index and the variations in the Dst index.

### 2.2. Station Selection

The stations were chosen based on data availability during the observed period and their location. Twenty-one stations from the International GNSS Service (IGS) network were selected. All selected stations are multi-GNSS (GPS, GLONASS, Galileo, and BeiDou) and provide data in Rinex 3.04 format or higher, ensuring compatibility with the software used in processing.

**Figure 5:**
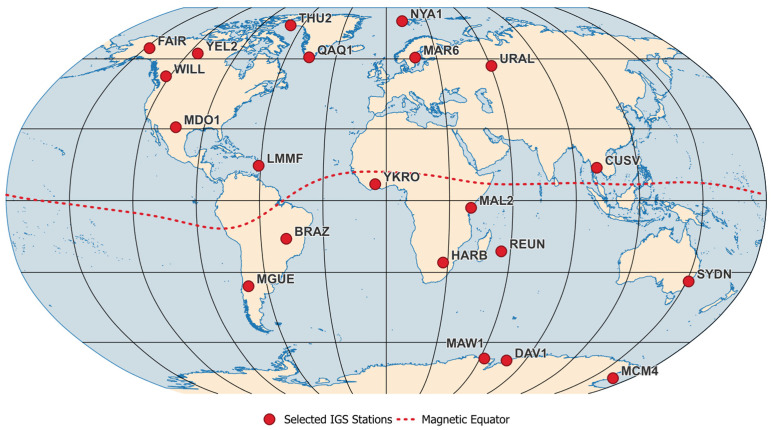
Map showing the locations of the selected stations.

Regarding station location, we opted for an equivalent global distribution by latitude band, divided into three groups of seven stations to cover low (between latitudes ±30°), medium (between latitudes ±30° to ±60°) and high (latitudes > |±60°|) latitude regions, with at least one station per continent. Figure 5 shows the location of the 21 selected stations and the geomagnetic equator.

This configuration is essential for investigating the different behaviors of the atmosphere, allowing the analysis of distinct phenomena: from the relative stability of the mid-latitudes to the severe effects of ionospheric scintillation and plasma bubbles in the equatorial regions, as well as the impact of geomagnetic storms at high latitudes. Additionally, this geographic sampling allows the capture of thermal and humidity variations that influence the refractive index of the neutrosphere, whose signal delay effects vary considerably across different climate zones.

**Figure 6:**
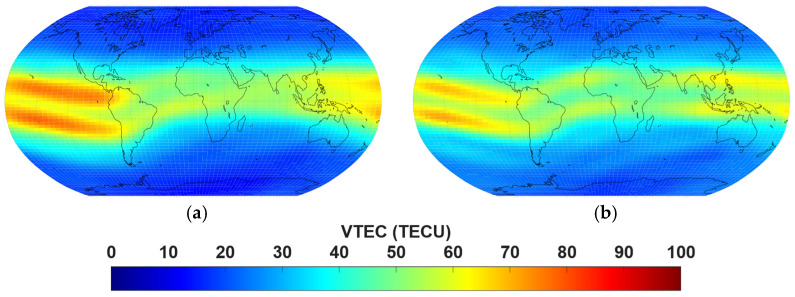
VTEC maps (in TECU) for days of high (**a**) and low activity (**b**).

Figure 6 shows the mean TEC during the days analyzed, including both high- and low-activity periods. When this quantity is projected vertically, it is referred to as Vertical TEC (VTEC). The data was obtained from IONEX files provided by the IGS. Higher electron concentrations are observed at low latitudes. During geomagnetically disturbed days, this concentration increases, reaching an average of 38.62 TECU (TEC Unit), as illustrated in Figure 6a. In contrast, on days of low activity, although enhanced electron concentrations persist in the equatorial region, the values are lower than those observed under disturbed conditions, with an average of 37.99 TECU, as shown in Figure 6b.

### 2.3. Processing Configurations

For the evaluation of this experiment, PPP processing was performed in an epoch-wise kinematic mode using static IGS reference stations. In this context, the term kinematic refers to a simulated kinematic strategy, in which the receiver coordinates are estimated independently at each epoch, rather than to observations collected by a moving platform. This configuration was adopted to assess the sensitivity of the positioning solution to atmospheric variability without imposing static coordinate constraints. The configurations used in the APPPOLO (Advanced Precise Point Positioning software for Optimized Localization) software [20] are shown in Table 2.

Signals from the four GNSS constellations were used, enabling better geometry and reducing positional dilution of precision (PDOP). With dual-frequency tracking, it was possible to perform ion-free combination for pseudorange ($p_{if}$) and carrier phase ($l_{if}$), in meters, given by [1]:

(1) $$p_{if}=\frac{f_{1}^{2}p_{1}-f_{2}^{2}p_{2}}{f_{1}^{2}-f_{2}^{2}}=\rho +dt_{r}+m_{f}ZWD+e,$$

(2) $$l_{if}=\frac{f_{1}^{2}l_{1}-f_{2}^{2}l_{2}}{f_{1}^{2}-f_{2}^{2}}=\rho +dt_{r}+m_{f}ZWD+N+e,$$

where the left side of the equations, given by the set of original observations of pseudorange $p_{1}$ and $p_{2}$ and phase $l_{1}$ and $l_{2\;}$, are derived from signals of frequency $f_{1}$ and $f_{2}$. Since the linear combination eliminates the first-order effects of the ionosphere, the right side of the equations, given by the linearized functional model of the observations, is represented by the geometric distance (ρ), receiver clock error ($dt_{r}$), the residual wet zenith component of the neutrosphere (zenith wet delay, ZWD), applying a mapping function $m_{f}$, and phase ambiguities N. The symbol *e* represents systematic error corrections for the observables, as well as their noise, thus also representing random effects.

To ensure that the analysis was not biased by the initial PPP convergence time, the combined smoothing method proposed by [21] was applied. This approach uses forward (from the beginning to the end) and backward (from the end to the beginning) estimates of the optimized parameters, based on their reliability as derived from the variance-covariance matrix (VCM). Cycle slips were detected using the TurboEdit algorithm proposed by [24].

**Figure 7:**
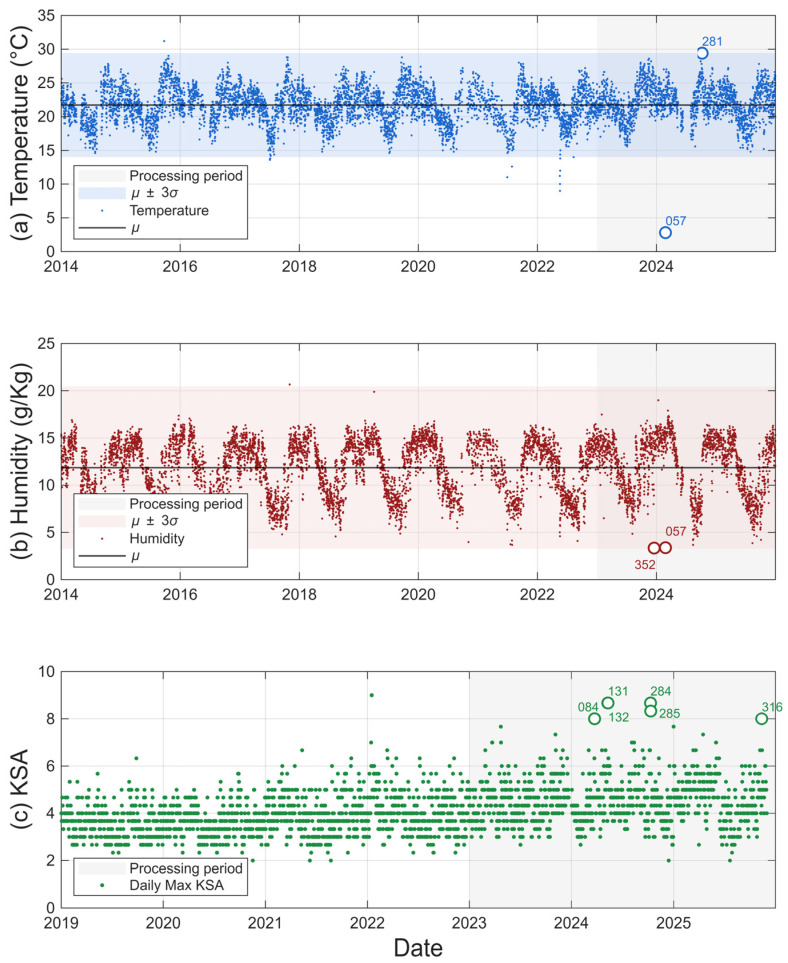
Selection of representative days for the assessment of neutral atmosphere correction models: (**a**) Time series of air temperature at the INMET A001 station, with highlighted days corresponding to extreme deviations from the mean (μ ± 3σ); (**b**) Time series of specific humidity, with selected days representing anomalous low humidity conditions; and (**c**) Daily maximum Ksa index over South America, with highlighted days indicating periods of enhanced ionospheric activity. Marker labels indicate day of the year (DOY).

**Figure 8:**
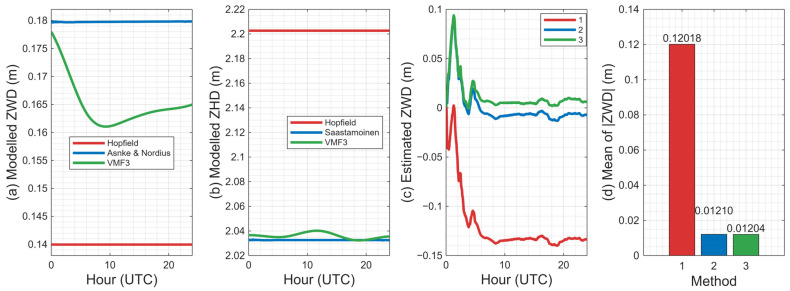
Time series of the mean values computed over the nine selected days for the BRAZ station (DF, Brazil), considering three neutral atmosphere modeling strategies: (**a**) Modeled ZWD; (**b**) Modeled zenith hydrostatic delay (ZHD); (**c**) Estimated ZWD; and (**d**) Mean absolute ZWD for each method. The three approaches correspond to Method 1, Method 2, and Method 3, as defined in Table 3.

The method used to correct neutrospheric effects was selected based on preliminary tests conducted prior to processing. Three days were selected as outliers in terms of temperature and pressure, observed using the NEPTool v1.0 [25] for the INMET A001 station (15.7894° S, 47.9258° W, 1160.96 m). Six more days were selected based on the South American Ksa index, provided by EMBRACE (https://www2.inpe.br/climaespacial/portal/pt/ (accessed on 20 January 2026)). These nine days were used exclusively for this preliminary assessment and are not part of the positioning experiment. Figure 7 shows the days selected along with neutrospheric parameters. Figure 8 shows time series of the ZTD (total zenith delay) estimate for the BRAZ station (approximately 18.2 km from INMET A001, with a height difference of about 55 m), using different neutral atmosphere modeling methodologies, as shown in Table 3. It is worth noting that kinematic positioning was performed in the forward direction. The other settings were preserved.

VMF3 was adopted because it is the model that keeps the ZWD estimate most stable and close to zero throughout the day (Figure 8c), while Method 1 shows a clear systematic deviation, with a persistent negative trend and a much higher average (≈0.120 m), indicating that the model does not adequately represent the atmospheric conditions of the period and forces the adjustment to “absorb” modeling error in the neutral atmospheric parameter; Method 3 produces much lower average ZWD values (approximately 0.012 m), comparable to Method 2, but with more consistent temporal behavior and no marked bias (Figure 8a,b), which implies better accuracy in ZTD data for neutrosphere analysis [23].

### 2.4. Evaluation Framework

The PPP solution analysis is performed based on positional accuracy, considering the bias of the geocentric positional components (x, y, z) and their respective uncertainties, using the root mean square error (RMSE). The coordinates taken as true ($r_{t}$) are derived from the multi-year solutions of the International Terrestrial Reference Frame 2020 (ITRF2020$,\;\;r_{0}$), updated for the processing epoch, by applying the linearized temporal variation:

(3) $$r_{t}=r_{0}+v_{0}dt,$$

where $v_{0}$ is the geocentric velocity of the station (m/year) and dt is the time interval, in years, between the GNSS observation epoch and the reference epoch of the ITRF2020 solution. The estimated ZWD is also analyzed, as well as its uncertainty, and used to calculate the ZTD. In addition, the residuals of the observables from each constellation are investigated and correlated with the level of ionospheric variability, elevation, and processing period. A comparison between the geometry and quality of the fit when using only GPS and the multi-constellation combination with the four constellations is also performed.

### 2.5. Ionospheric Irregularity Indexes

Ionospheric irregularities can be assessed through indexes that quantify the variability of the TEC, such as the Rate Of TEC Index (ROTI). ROTI is derived from the ROT (Rate Of TEC), which represents the temporal rate of change of TEC and can be calculated by [31]:

(4) $$ROT=\frac{\Delta \mathrm{TEC}}{\Delta t}$$

where ΔTEC is the TEC difference obtained from the difference between two consecutive epochs, and Δ*t* is the time interval between the measurements.

High ROTI values indicate greater variability in TEC, generally associated with the presence of ionospheric irregularities. The ROTI is calculated from the standard deviation of ROT within a five-minute interval using the following equation [32]:

(5) $$ROTI=\sqrt{\left\langle {ROT}^{2}\right\rangle -{\left\langle ROT\right\rangle }^{2}}$$

where ⟨ROT⟩ is the arithmetic mean of ROT during the measured interval, and ⟨ROT^2^⟩ corresponds to the mean of the squared ROT values.

**Figure 9:**
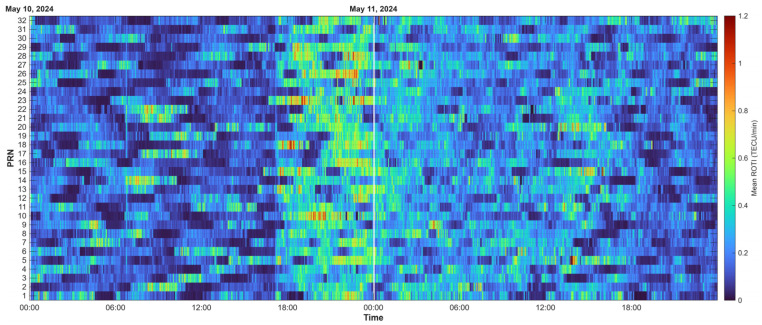
Temporal distribution of the mean ROTI for each GPS PRN between 10 and 11 May 2024.

The analysis of the ROTI was conducted using the global average per satellite over the selected study period, focusing specifically on 10 and 11 May 2024, which were adopted as case study days. It should be emphasized that ROTI was not used as an input in the PPP processing, nor was it incorporated into the functional or stochastic models. Instead, it was computed independently and used as an external diagnostic metric to characterize ionospheric irregularities and support the interpretation of PPP performance under disturbed geomagnetic conditions. Figure 9 presents the temporal distribution of the average ROTI for different GPS satellites (PRNs), computed from dual-frequency L1/L2 observations using CODE differential code bias (DCB) products (P1–C1 and P1–P2). Among these, 10 May corresponds to the most severe day of the analyzed interval and is associated with the geomagnetic storm widely known as the “Mother’s Day Geomagnetic Storm”, allowing for a comparative assessment of ionospheric conditions under disturbed scenarios [33].

It can be observed that between approximately 18:00 and 00:00 UTC (Universal Time Coordinate) there is a significant increase in the index. This behavior is consistent with the occurrence of ionospheric irregularities during the post-sunset period and the occurrence of a geomagnetic storm. The presence of these phenomena causes greater variability in TEC, which is reflected in the elevated ROTI values observed simultaneously across multiple PRNs.

**Figure 10:**
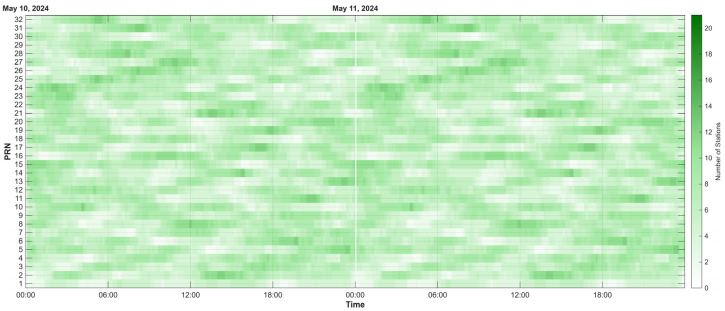
Number of GNSS stations used in the calculation of the ROTI for each GPS PRN throughout 10 and 11 May 2024. Although 21 globally distributed stations were considered in the analysis, the number of stations varies by PRN and epoch according to satellite visibility and data availability.

Figure 10 shows the number of stations used to calculate the ROTI for each PRN throughout 10 and 11 May. Although the analysis was based on a network of 21 GNSS stations distributed worldwide, the number of stations contributing to each PRN is not necessarily equal to 21 at every epoch. This occurs because each station observes only the satellites that are visible above its local horizon and that satisfy the adopted data availability and quality criteria. Therefore, different PRNs may be tracked by different subsets of stations at a given time. It can be observed that during the period when the significant increase in the ROTI occurs in Figure 9, the number of available stations remains relatively stable for most PRNs.

This behavior indicates that the high ROTI values observed during this interval are not associated with a reduction in the number of stations used in the calculation, but rather with a real increase in the variability of TEC in the ionosphere. Therefore, the consistency in the number of stations reinforces the interpretation that the increase in ROTI is related to the occurrence of ionospheric irregularities during the post-sunset period, which coincides with the time of high solar activity and geomagnetic storm.

### 2.6. Adoption of an Appropriate Stochastic Model

While the functional model describes the deterministic component of the observations, the stochastic model describes the randomness of the system’s dynamics, thus incorporating uncertainty [34]. Assuming no correlation between the observables, given that there is no differentiation between them, the covariance matrix of the observations can be represented in diagonal form. The application of ion-free leads to noise amplification, an inherent characteristic of combining observables, and can be described for observations from s satellites by:

(6) Σy=f12f12−f22⏞m1f22f12−f22⏞m20000m1m2⋮⋮⋮⋮m1m20000m1m2σp112    0 σp212      σl112      σl222      ⋱ 0    σl2s2m10⋯m10m20⋯m200m1⋯0m10m2⋯0m2,

where $\Sigma_{y}$ is the covariance matrix of the observations. The a priori uncertainty $\sigma_{0}$ adopted for the pseudorange observables was 0.8 m and 1 m, while the phase uncertainty is 0.008 m and 0.010 m for the first and second frequencies, respectively. A weighting based on the constellation geometry, i.e., on the elevation E of each satellite, is applied, thus benefiting those closest to zenith. Therefore, given an observation y, its uncertainty is given by:

(7) $$\sigma_{y}=\sigma_{0}csc\;E.$$

In recursive PPP processing, the observations were adjusted using the Robust Adaptive Kalman Filtering, in which the estimated parameters receive different stochastic treatments according to their expected dynamics over time. The receiver coordinates are kept constant in static processing, while in kinematic mode they vary at each epoch and are modeled as white noise. The receiver clock was also treated as white noise to accommodate abrupt variations and occasional reboots. The ZWD was modeled as a random walk, representing its gradual evolution over time. Ambiguities were kept constant when there were no cycle slips, but reset when necessary, ensuring consistency throughout processing and contributing to efficient quality control of the solution. For more details, see [20].

In addition to the stochastic modeling adopted, recursive processing was complemented by a quality control procedure based on the Detection, Identification, and Adaptation (DIA) paradigm, applied locally to each epoch. Initially, a global statistical test is performed to verify the consistency of the model based on the predicted residuals, assuming a zero mean under nominal conditions. For this purpose, the Local Overall Model (LOM) statistics are used, calculated by [35]:

(8) $$LOM=\frac{1}{n_{obs}}V^{t}\Sigma_{V}^{-1}V,$$

where $n_{obs}$ represents the number of observations for the period, V is the vector of predicted residuals, and $\Sigma_{V}$ is the associated covariance matrix. When the LOM indicates inconsistency, an identification step is applied to locate the observation most likely to be contaminated, using a Baarda-style data snooping criterion. Finally, the adaptation step mitigates the influence of the detected error by reweighing the observations and/or resetting the affected parameters, preventing the propagation of outliers and increasing the reliability of the PPP solution throughout the processing chain.

## 3. Results

This section is organized into five parts. The first analyzes the average number of tracked satellites and the corresponding geometry quality on a global scale. The second focuses on the total neutrospheric delay, presenting the spatial and temporal behavior of the ZHD and ZWD based on the VMF3 grid values. The third addresses the impact of ionospheric disturbances, including the characterization of scintillation effects using the ROTI. The fourth evaluates the positional accuracy obtained from the 924 processing runs, discussing deviations from the theoretical trajectory under a simulated kinematic scenario, as well as the improvements associated with the inclusion of multiple constellations. Finally, the section concludes with an analysis of the residuals of ionosphere-free observables under both quiet and disturbed geomagnetic conditions.

### 3.1. Satellite Geometry and Visibility

**Figure 11:**
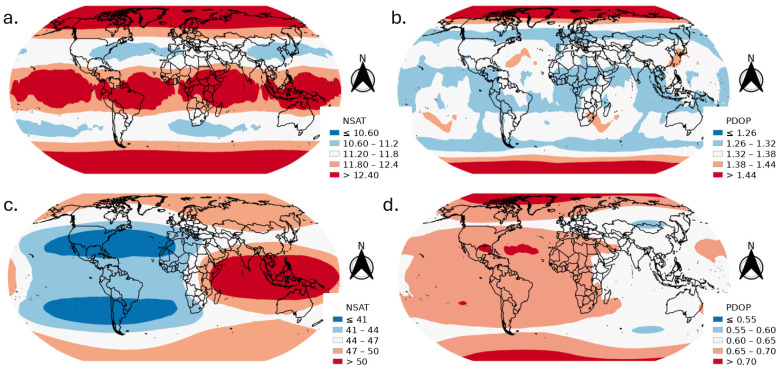
Global distribution of mean Nsat and PDOP values computed over all selected days for GPS-only processing (**a**,**b**) and the multi-GNSS solution (**c**,**d**).

Precise CODE ephemerides were used to perform this analysis. The number of tracked satellites (Nsat) and PDOP were calculated on a 2° × 2° grid. The improvement in satellite availability and PDOP when using multi-GNSS instead of GPS-only is expected and is presented here to provide context for interpreting how constellation configuration, together with geomagnetic disturbance level, latitude band, satellite elevation angle, and ROTI behavior, influences PPP accuracy and stability. Figure 11 summarizes, on a global scale, how satellite availability and observational geometry change when comparing the use of a single constellation (a, b) with the multi-GNSS solution employing the four global systems (c, d), using maps of the average Nsat and PDOP for the sampled days.

It can be observed that, when restricting processing to GPS, the average visibility typically remains in the range of ~10–12 satellites, with PDOP around ~1.26–1.44, which is consistent with the computed averages (Nsat = 12.2; PDOP = 1.4), while in the multi-constellation configuration, there is a significant and spatially more homogeneous increase in availability (typically > 41 satellites), accompanied by a systematic reduction in PDOP to values close to 0.5–0.7, reflected in the overall average (Nsat = 46.1; PDOP = 0.7). These results confirm the expected geometric advantage of multi-GNSS processing. In the context of this study, this advantage is interpreted as a supporting factor for the subsequent analyses, since increased observational redundancy and improved geometric strength can mitigate, but not eliminate, PPP degradation under disturbed ionospheric conditions. BeiDou also contributes to the multi-GNSS geometry through its mixed orbital architecture, composed of medium Earth orbit (MEO), inclined geosynchronous orbit (IGSO), and geostationary orbit (GEO) satellites. This diversity adds complementary viewing directions and helps stabilize the global PDOP distribution when combined with GPS, GLONASS, and Galileo.

### 3.2. Total Neutrosphere Delay

**Figure 12:**
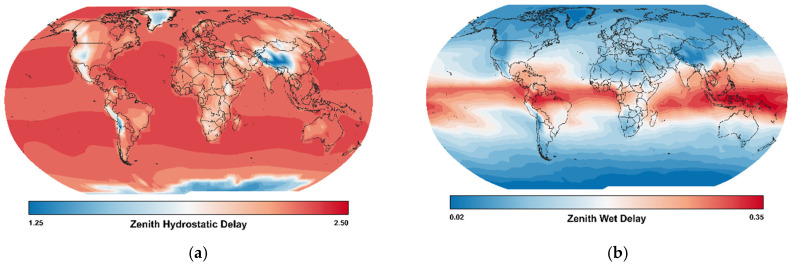
Global mean fields of (**a**) ZHD and (**b**) ZWD, in meters, obtained from the VMF3 grid over the 22 days of processing.

Figure 12 shows the average ZHD and ZWD fields obtained from the VMF3 grid values over the 22 days analyzed. ZHD exhibits variation predominantly controlled by pressure/altitude, with lower values over regions of high topography and higher values at low altitudes, reflecting the highly deterministic nature of the hydrostatic component. In contrast, ZWD shows a latitudinal pattern associated with water vapor distribution, with maximums concentrated in the tropical/equatorial range and minimums at higher latitudes, consistent with the more variable and less predictable nature of the wet term.

**Figure 13:**
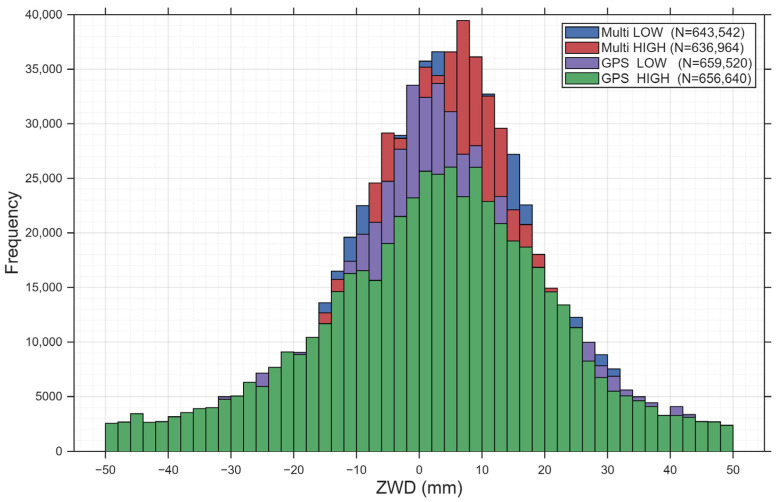
Histogram of the residual component of the estimated ZWD, comparing multi-GNSS and GPS-only solutions in the two subsets of days (lower and higher geomagnetic activity).

Figure 13 shows the histogram of the residual component of the ZWD estimated in the Kalman filter, modeled as a random walk process, comparing the multi-GNSS and GPS-only solutions in two subsets of days (11 days of lower and higher geomagnetic activity each). The distributions are predominantly unimodal, with tails indicating less frequent episodes of greater magnitude. A greater concentration of the histogram is observed in multi-GNSS, consistent with increased observational redundancy and greater stability in the estimation of the humid parameter, while in GPS-only the distribution tends to be more dispersed. The differences between the subsets of days are interpreted as an indirect effect of processing conditions, since geomagnetic activity does not directly influence the wet neutrosphere delay.

### 3.3. Degradation of PPP Due to Ionospheric Scintillation

**Figure 14:**
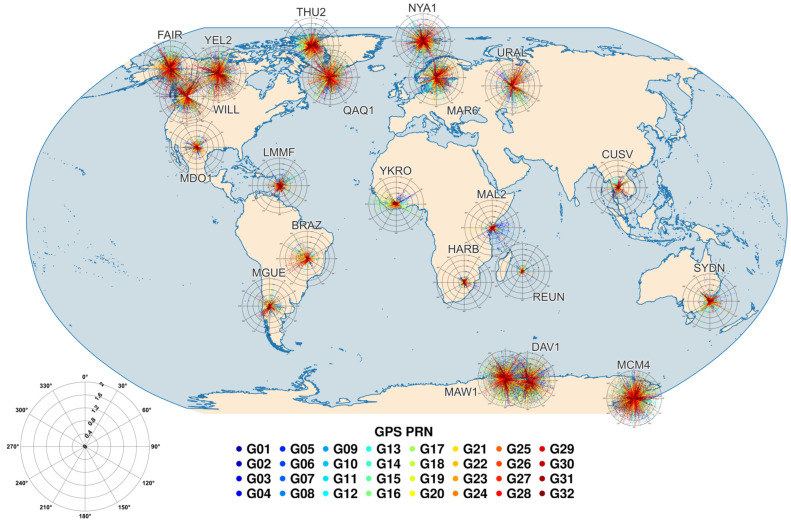
Global distribution of the ROTI derived from GNSS observations at different stations during the period from 10 to 11 May 2024.

Figure 14 shows the global distribution of the ROTI (ROTI × azimuth) for each station during 10 and 11 May 2024. It can be observed that the highest ROTI values are concentrated in high-latitude regions, especially near the poles, while lower values prevail in mid-latitudes.

This behavior occurs due to variations in ionospheric conditions across different geomagnetic regions. In high latitudes, the ionosphere exhibits more unstable behavior mainly during geomagnetic storm conditions, when enhanced ionospheric scintillation affects both the phase and amplitude of GNSS signals [32]. The large TEC variations observed throughout the study period, reflected in increased ROTI values, are consistent with these disturbed ionospheric conditions. In contrast, low-latitude regions present ROTI peaks at specific local times; however, the average value remains lower.

From the PPP perspective, this distribution implies that receivers located at high latitudes are more susceptible to positioning degradation due to the greater variability of the GNSS signal.

**Figure 15:**
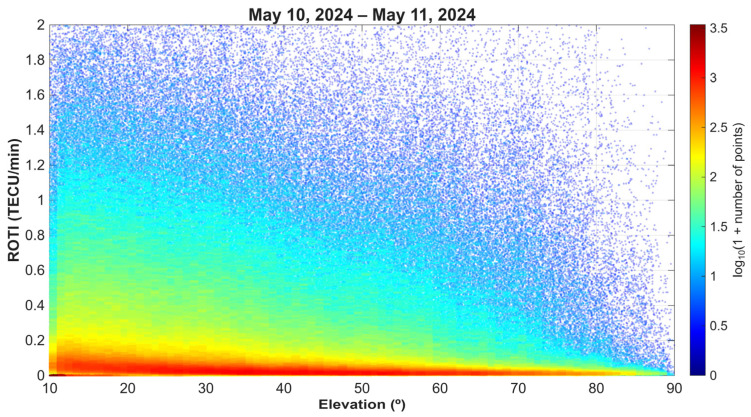
Distribution of the ROTI as a function of GNSS satellite elevation angle for the period from 10 to 11 May 2024.

Figure 15 shows a graph of ROTI as a function of satellite elevation angle for all stations, revealing an inversely proportional relationship between these variables for both analyzed days. High ROTI values are mainly concentrated at low elevation angles (10–30°), whereas for angles above 70°, a significant reduction is observed in both the magnitude and variability of ROTI.

This pattern can be mainly explained by the geometry of the GNSS signal path through the ionosphere. At low elevation angles, the signal travels a longer oblique path through the ionosphere, increasing the probability of intersecting multiple electron density irregularities. This results in greater TEC fluctuations and, consequently, higher ROTI values.

For PPP, this behavior has direct implications: low-elevation observations tend to introduce more noise into GNSS observables, potentially compromising the convergence and stability of the solution. This justifies the adoption of more restrictive elevation masks in environments subject to scintillation.

The analysis of ROTI by azimuthal quadrants reveals distinct spatial patterns (Figure 14). High-latitude stations present the highest average ROTI values, with MCM4 (0.5434), DAV1 (0.5248), and MAW1 (0.5035) indicating enhanced ionospheric irregularity. In contrast, mid-latitude stations exhibit moderate ROTI values, such as MAR6 (0.2786), THU2 (0.2657), and URAL (0.2314). At low latitudes, stations such as CUSV (0.0670), HARB (0.0493), and REUN (0.0456) present significantly lower ROTI values.

Overall, PPP degradation during ionospheric scintillation events is controlled by spatial (latitude, direction), geometric (elevation), and temporal (storm intensity) factors. Understanding these patterns is essential for improving GNSS positioning robustness under disturbed ionospheric conditions.

### 3.4. Accuracy of Kinematic Positioning

**Figure 16:**
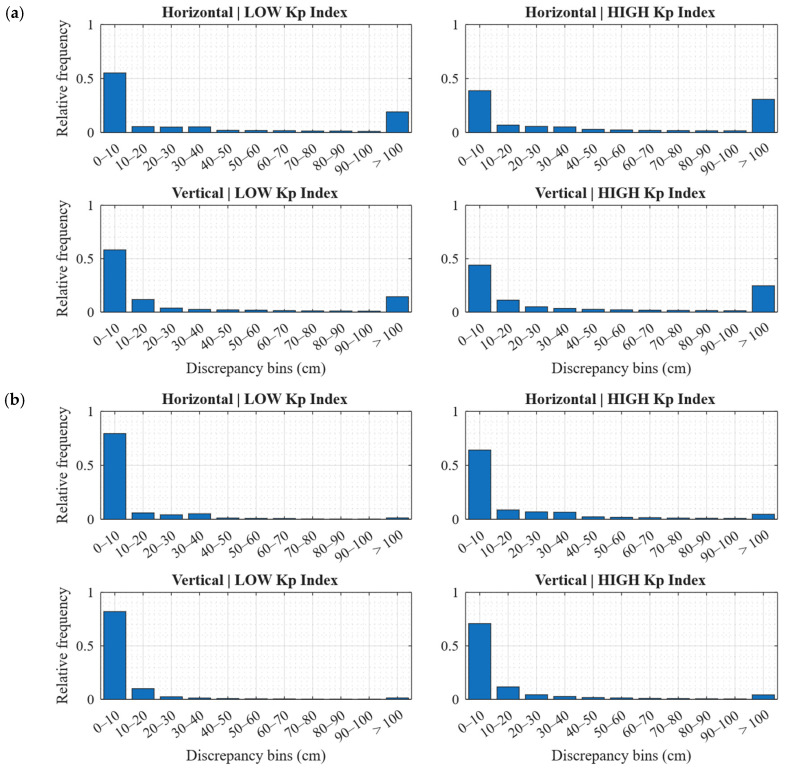
Histogram of discrepancies for kinematic PPP using data from the (**a**) GPS and (**b**) multi-GNSS constellations.

Figure 16 presents the relative frequency distribution of the kinematic PPP discrepancies in different discrepancy bins, separating the horizontal and vertical components and two levels of geomagnetic activity. The histograms are shown for GPS-only and multi-GNSS processing, allowing the comparison of how the discrepancies are distributed under low and high Kp index conditions.

For the multi-GNSS solution, the highest concentration in the 0–10 cm range occurs at low latitudes, with percentages above 89% in both components (reaching 95.28% in the horizontal and 92.76% in the vertical in the most disturbed scenario). At mid-latitudes, this concentration decreases, especially in the horizontal plane, ranging from 56.89% to 48.23%, while the vertical component remains relatively more preserved (79.73% to 70.46%). At high latitudes, sensitivity to environmental conditions becomes more evident: the fraction in 0–10 cm goes from 78.76% (H) and 77.15% (V) to 46.62% (H) and 53.67% (V) when considering the period of greatest activity. For GPS processing, the percentages in the 0–10 cm range are systematically lower than those for multi-GNSS in all bands, with marked degradation at mid and, especially, high latitudes under more severe conditions. At high latitudes, the fraction in 0–10 cm is reduced to 20.14% horizontally and 27.28% vertically, indicating a predominance of discrepancies above the centimeter level. Although low-latitude regions are commonly affected by the EIA and by ionospheric plasma bubbles, the impact of geomagnetic storms on PPP performance in these regions is not necessarily uniform. The development of post-sunset irregularities depends on local time, storm phase, prompt penetration electric fields, and disturbance dynamo effects. In the analyzed events, the disturbed dynamo may have partially inhibited the formation or intensification of ionospheric irregularities during specific periods, which helps explain the relatively stable behavior observed at low latitudes. Therefore, the high concentration of discrepancies within the 0–10 cm range should be interpreted as an event-dependent result, also favored by the ionosphere-free combination, robust quality control, and the increased observational redundancy of the multi-GNSS solution.

**Figure 17:**
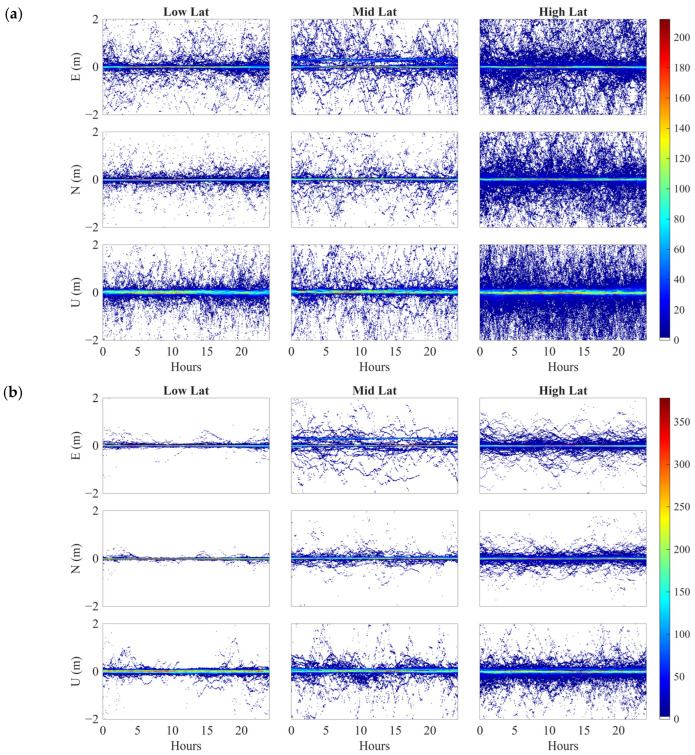
Density maps of East (E), North (N), and Up (U) discrepancies throughout the day for each latitudinal band, considering all processed days and all stations within each band: (**a**) GPS-only and (**b**) multi-GNSS solutions.

Figure 17 shows the daily evolution of discrepancies in the E, N, and U components, by latitudinal band, simultaneously highlighting the density around zero and the spread over 24 h. For multi-GNSS solutions, predominantly centimeter-level behavior is observed at low latitudes, which also reflects the better geometry of satellites in regions close to the equator, as discussed in Section 3.1, due to the greater availability of satellites and the more favorable spatial configuration of the constellations. The averages were 5.59 cm (H) and 9.11 cm (V) in the most stable scenario and 4.14 cm (H) and 6.32 cm (V) in the most disturbed scenario, consistent with the strong concentration of density close to zero throughout the day. At mid-latitudes, dispersion increases, with averages rising to 17.47 cm (H) and 10.26 cm (V) and reaching 27.93 cm and 21.58 cm under less favorable conditions. At high latitudes, the effect is even more pronounced, with averages reaching 32.94 cm (H) and 43.08 cm (V) in the most severe scenario, accompanied by a higher recurrence of decimeter and meter-level discrepancies in the three components. In the GPS-only solution, the averages remain significantly higher in all latitudinal ranges, and the spread is more persistent throughout the day, consistent with the weakening of the concentration around zero observed in the maps. Even at low latitudes, the averages remain in the order of tens of centimeters (53.82 cm horizontally and 69.13 cm vertically), and at high latitudes, in the most severe scenario, they reach 138.84 cm (H) and 154.93 cm (V), characterizing a more frequent occurrence of discrepancies greater than 1 m.

### 3.5. Quality Control

**Figure 18:**
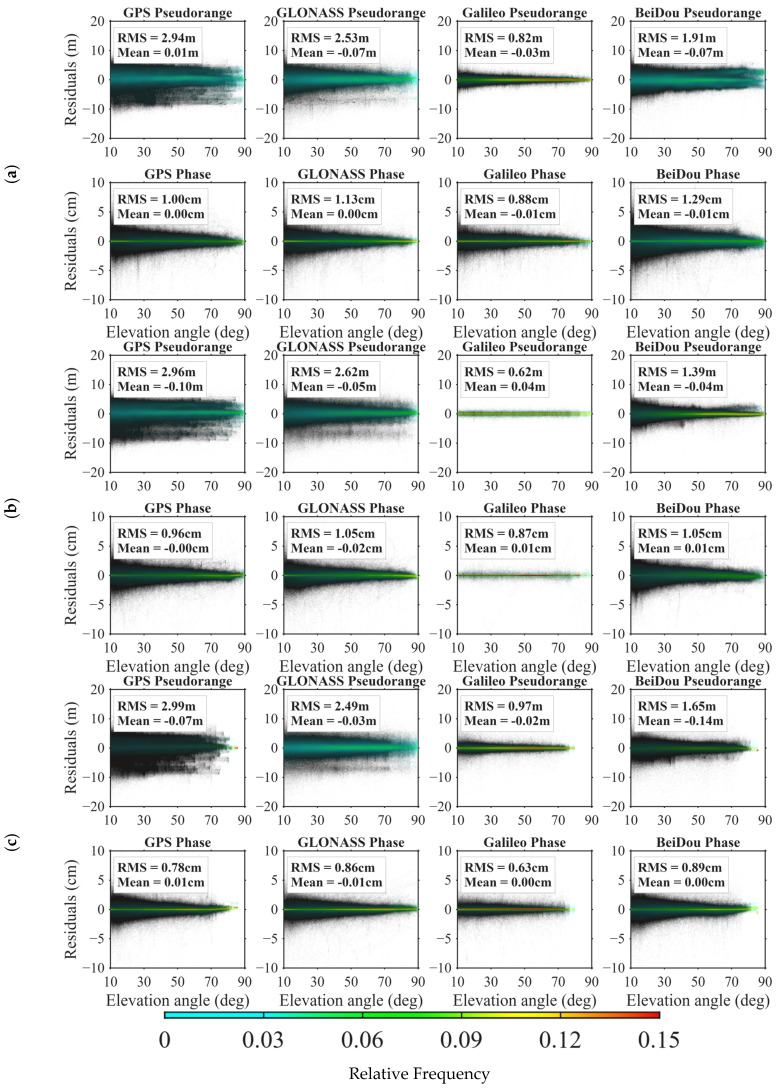
Residuals of pseudorange and carrier phase observations as a function of satellite elevation for the low Kp index scenario, separated by latitude bands: (**a**) Low, (**b**) Medium, and (**c**) High.

**Figure 19:**
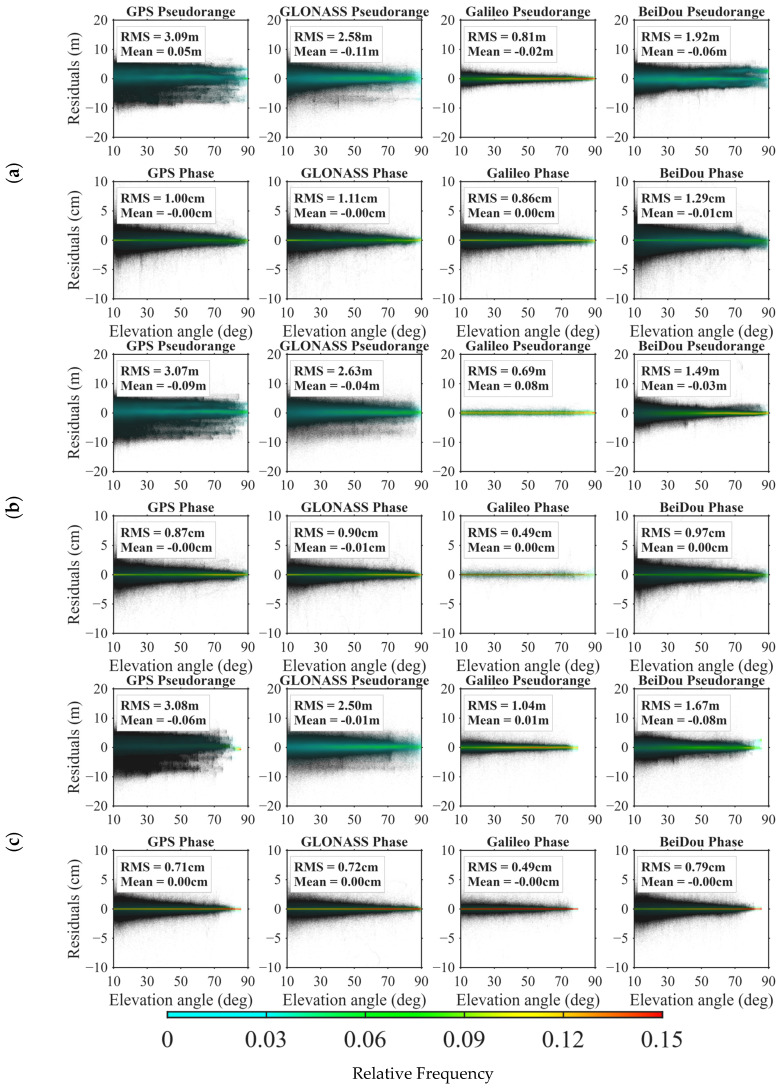
Residuals of pseudorange and carrier phase observations as a function of satellite elevation for the high Kp index scenario, separated by latitude bands: (**a**) Low, (**b**) Medium, and (**c**) High.

Figure 18 and Figure 19 show the post-adjustment residuals as a function of elevation angle for pseudorange and carrier phase observations, segregated by constellation and latitude bands. In all configurations, there is a strong dependence on elevation, with greater dispersion at low elevations and progressive contraction of the residual cloud as the satellites approach the zenith.

The average residual remains close to zero for both pseudorange and phase, indicating no relevant systematic bias after adjustment. In terms of Root Mean Square (RMS), pseudorange shows marked differences between constellations, with GPS and GLONASS concentrating the highest values (≈3 m and ≈2.5–2.6 m), BeiDou at an intermediate level (≈1.4–1.9 m), and Galileo exhibiting the most compact distribution (≈0.6–1.0 m). For carrier phase, the residuals remain at the centimeter level, with Galileo again standing out, and variations between scenarios compatible with the combined effect of elevation weighting and quality control, which tends to mitigate the contribution of more degraded observations in the effectively adjusted set.

**Figure 20:**
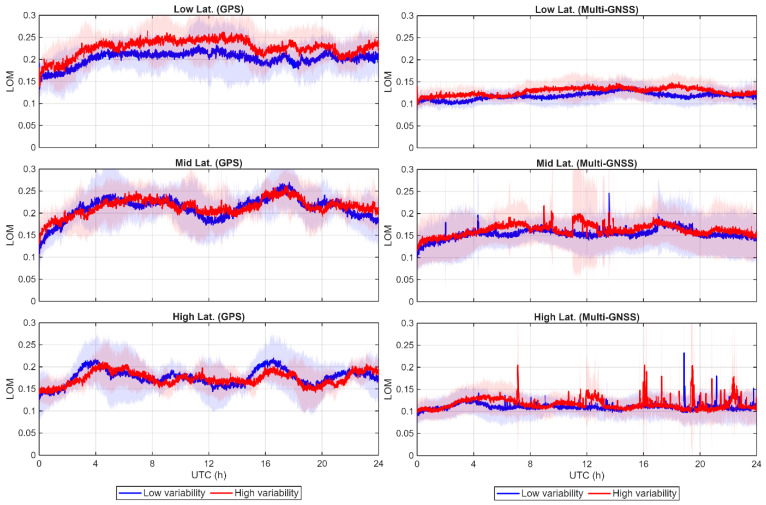
Time series of the local test statistic (LOM) by latitudinal band for multi-GNSS and GPS-only solutions under low and high variability. The curves represent the average value throughout the day, and the shaded bands indicate the intraday dispersion of the LOM.

Figure 20 shows the temporal evolution of the LOM test statistics by latitudinal band, comparing multi-GNSS processing and the GPS-only scenario under low and high variability conditions. It can be observed that multi-GNSS maintains lower average LOM values and reduced dispersion throughout the day, indicating a more consistent adjustment. In the GPS-only case, the LOM operates at a higher level and with greater variability, with a more evident separation between the two conditions, particularly at low latitudes. At medium and high latitudes, the difference between the curves is reduced, and convergence and crossing intervals are observed, indicating that the daily geometry dynamics and the effective set of observations play a relevant role in the test response. It should be noted that the LOM statistics are evaluated against a chi-square threshold defined at a 1% significance level, with the acceptance criterion depending on the number of observations in each class (degrees of freedom). Therefore, no single fixed threshold applies uniformly over time, and variations in the LOM values must be interpreted considering changes in observational geometry and redundancy. The lower LOM values observed in the multi-GNSS solution are not solely a consequence of the increased number of observations but also reflect improvements in the overall quality and consistency of the adjustment. In particular, the addition of new constellations increases the degrees of freedom of the system and contributes to a more stable stochastic behavior, which directly impacts the normalized test statistic.

## 4. Conclusions

This work presented a multi-scale approach to evaluate, throughout Solar Cycle 25, the impacts of the ionosphere and neutrosphere through an independent analysis of each component on PPP accuracy and stability, comparing processing with a single constellation (GPS-only) and with a multi-GNSS solution. The strategy adopted, based on the selection of geomagnetically calm and disturbed days via the Kp index and on global analysis with 21 multi-GNSS stations, allowed for the systematic investigation of spatial (by latitude bands) and temporal (intraday dynamics) effects, totaling 924 processes (462 per scenario).

In the kinematic PPP evaluation, degradation under severe geomagnetic conditions exhibited a clear latitude dependence, with performance deteriorating most at high latitudes. Multi-GNSS processing consistently outperformed GPS-only solutions across all latitude bands, concentrating discrepancies primarily within 0–10 cm and markedly reducing decimeter- to meter-level errors. This improvement reflects increased satellite availability, improved observation geometry, and greater redundancy in the adjustment. Residual analysis showed post-adjustment behavior consistent with the adopted elevation weighting and quality-control strategy, with mean residuals near zero. Pseudorange residuals displayed larger, constellation-dependent differences, while carrier-phase residuals remained at the centimeter level. The LOM test confirmed the superior consistency of the multi-GNSS adjustment, yielding lower average values and reduced intraday dispersion.

As for the neutrosphere delay, the mean fields derived from VMF3 reinforced the more deterministic nature of the hydrostatic term (ZHD) and the latitudinal variability of the wet term (ZWD), with maxima in the tropical/equatorial range. The residual component of the ZWD estimated in the filter showed a more concentrated distribution in the multi-GNSS, indicating greater stability in the estimation of the wet parameter due to increased observational redundancy, while the differences between subsets of days were interpreted as indirect, since geomagnetic activity does not act directly on the wet delay. Additionally, the assessment of ionospheric irregularities using the ROTI indicates that PPP performance degradation is closely linked to both spatial distribution and satellite geometry. Enhanced irregularities, more pronounced at high latitudes during disturbed periods, increase the vulnerability of GNSS positioning. In addition, as expected, the observed dependence on satellite elevation shows that signals at lower angles are more affected, as they traverse longer paths through the ionosphere, introducing additional noise and reducing solution stability and convergence. These findings highlight the need to account for scintillation effects in PPP strategies, particularly under challenging ionospheric conditions, to improve positioning reliability.

As a prospect for future work, we propose conducting a case study focused on the development of a global interpolated ROTI grid, enabling a continuous spatial representation of ionospheric irregularities based on distributed GNSS observations. In this context, the estimation of the ROTI should be extended to a multi-frequency, multi-constellation framework, rather than being limited to the traditional L1/L2 combination, allowing a more comprehensive and robust characterization of ionospheric disturbances. Such an approach would support a more detailed representation of scintillation patterns worldwide, improving modeling and mitigation strategies in PPP applications. In addition, this framework could be extended to real-time PPP simulations, in which the interpolated ROTI derived from multi-frequency and multi-constellation observations, would be incorporated as an external indicator of signal quality, enabling dynamic weighting, detection, and exclusion of degraded observations. This perspective opens new possibilities for enhancing PPP robustness, particularly in operational contexts subject to adverse ionospheric conditions.

## Figures and Tables

**Table 1:** Selected Days.

Disturbed Days	Calm Days
24 March 2023	27 March 2023
23 April 2023	3 May 2023
24 April 2023	16 April 2023
24 March 2024	17 March 2024
10 May 2024	4 May 2024
11 May 2024	9 May 2024
12 August 2024	15 August 2024
10 October 2024	13 October 2024
11 October 2024	21 October 2024
1 January 2025	26 December 2024
12 November 2025	19 November 2025

**Table 2:** Processing settings.

Parameters	Strategy
Observables	GPS: L1 C/A, L2 P(Y); GLONASS: G1, G2; Galileo: E1, E5a; BeiDou: B1, B3.
Estimator	Forward-Backward Smoothing [21] + Robust Adaptive Kalman Filtering [22].
Orbits and Clock	Precise Ephemeris SP3 and CLK files from CODE (Center for Orbit Determination of Europe).
Neutrosphere Effects	Mapping Function, wet zenith delay and hydrostatic zenith delay obtained by VMF3 + Neutrospheric Gradients (GPT3) [23] + residual component estimated as random walk.
Ionospheric Effects	Eliminated by the ionosphere-free linear combination (ion-free).
Coordinates	Estimated as white noise.
Other systemic effects	Wind-up, Sagnac, PCO (Phase Center Offset), PCV (Phase Center Variation), OSB (Observable Specific Bias), terrestrial and ocean tides, and relativistic effect on clock and distance.
Ambiguity	Estimated as constants (white noise when cycle slip occurs).

**Table 3:** Description of the experiment’s neutrosphere modeling settings.

Method	Mapping Function	Hydrostatic	Wet	Gradient
1	Niell [26]	Hopfield [27]	Hopfield	-
2	GMF [28]	Saastamoinen [29]	Askne & Nordius [30]	GPT3 [23]
3	VMF3 [23]	VMF3	VMF3	GPT3

## Data Availability

The GNSS data and products used in this study are publicly available. The multi-GNSS observation data were obtained from the IGS (<https://www.igs.org> (accessed on 15 January 2025)). Precise satellite orbit and clock products were provided from the IGS (<https://www.igs.org> (accessed on 15 January 2025)) and the CODE (<https://www.aiub.unibe.ch> (accessed on 15 January 2025)), while OSB and additional products were provided by Wuhan University (<https://en.whu.edu.cn/> (accessed on 15 January 2025)). Ksa index was provided by EMBRACE (<https://www2.inpe.br/climaespacial/portal/pt/> (accessed on 20 January 2026)). Kp index was provided by GFZ (<https://kp.gfz.de/en/> (accessed on 25 February 2026)). The Dst index used in this paper/presentation was provided by the WDC for Geomagnetism, Kyoto (https://wdc.kugi.kyoto-u.ac.jp/wdc/Sec3.html (accessed on 25 February 2026)). NEPTool (<https://neptool.fct.unesp.br/> (accessed on 20 January 2026)). Sunspot Number data was provided by SILSO (<https://www.sidc.be/SILSO/home> (accessed on 25 February 2026)). ROTI was calculated using CSSRG MATLAB-TEC calculation R2.04 v.1.2 source code. This program created by Excellence Center in GNSS and Space Weather, Thailand (<https://iono-gnss.kmitl.ac.th/> (accessed on 18 March 2026)).
